# Reduced HMGB 1-Mediated Pathway and Oxidative Stress in Resveratrol-Treated Diabetic Mice: A Possible Mechanism of Cardioprotection of Resveratrol in Diabetes Mellitus

**DOI:** 10.1155/2016/9836860

**Published:** 2016-10-19

**Authors:** Han Wu, Zhen-Qiang Sheng, Jun Xie, Ran Li, Liang Chen, Guan-Nan Li, Lian Wang, Biao Xu

**Affiliations:** ^1^Department of Cardiology, Drum Tower Hospital, Nanjing University Medical School, Nanjing 210008, China; ^2^Department of Cardiology, Drum Tower Clinical Medical Hospital, Nanjing Medical University, Nanjing 210008, China; ^3^Department of Cardiology, The Second Affiliated Hospital of Nantong University, Nantong 226001, China

## Abstract

Myocardial fibrosis and inflammation are intricately linked in diabetic cardiomyopathy (DCM), and resveratrol has been shown to attenuate oxidative stress, inflammation, and fibrosis in several cell types or animal models. High mobility group box 1 (HMGB 1), a proinflammatory cytokine, has been reported to regulate fibrosis and inflammation in various organs. Then the present study aimed to reveal the expression of HMGB 1-mediated signaling pathway and oxidative stress in resveratrol-treated diabetic mice. The significant increase in serum HMGB 1 concentration in diabetic mice was attenuated by treatment with resveratrol. Similarly, western blot analysis revealed a significant increase of HMGB 1 protein in monocytes and heart tissues of diabetic mice, and resveratrol partly normalized the changes. In addition, resveratrol abrogated the increased expression of HMGB 1-mediated signaling pathway, oxidative stress, fibrosis, and inflammation in diabetic hearts. In conclusion, inhibition of HMGB 1-mediated signaling pathway and oxidative stress may contribute to resveratrol-induced anti-inflammatory and antifibrotic effects in DCM.

## 1. Introduction

The leading causes of mortality in patients with diabetes mellitus (DM) are cardiovascular complications including hypertension, coronary heart disease, and diabetic cardiomyopathy (DCM) [1, 2]. The latter complication is a complex condition characterized by both early-onset diastolic and late-onset systolic dysfunctions without hypertension and coronary heart disease [3]. Epidemiological studies have shown that DCM is a major etiologic factor contributing to heart failure [4]. Until recently, the mechanisms involved in the pathogenesis of DCM remain incompletely understood; however, numerous mechanisms have been proposed including oxidative stress, inflammation, and fibrosis [5, 6].

Resveratrol, a polyphenolic compound and naturally occurring phytoalexin present in red wine and vegetable foods, has been shown to delay the progression of DCM [7–10]. Many pieces of evidence have implicated that the cardioprotection of resveratrol was, in part, due to its antioxidative effect. However, the precise cellular and molecular mechanism underling resveratrol-induced cardioprotection in DCM is still far from being fully elucidated.

High mobility group box 1 (HMGB 1) is a highly conserved nuclear protein present in various cells including eukaryotic cells [11]. A considerable number of studies have indicated upregulated HMGB 1 in various diseases including heart failure, stroke, and severe sepsis. Since convincing in vitro and in vivo evidence suggest that HMGB 1 increases in patients with DM or high glucose condition [12–15], HMGB 1 may be a novel cytokine contributing to pathological processes of DCM. Recently, the importance of HMGB 1 in DCM was also documented in studies utilizing genetic inhibition of HMGB 1, thus mitigated cardiac fibrosis and remodeling in DCM [16]. Additionally, Delucchi et al. found that resveratrol partly but significantly inhibited the upregulated HMGB-1 levels in diabetic rats [10]. It appears that HMGB-1 may be a therapeutic target for resveratrol in DCM; however, the underlying mechanism remains poorly understood.

HMGB 1 exerts its proinflammatory effect via binding to receptor for advanced glycation end products (RAGE) and toll-like receptors (TLRs) [11]. Binding of HMGB 1 to RAGE or TLRs leads to NF-*κ*B activation, which is known to evoke inflammatory response [11]. In addition to inflammation, HMGB 1 plays an important role in fibrogenesis [17–19]. However, there is little information regarding HMGB 1-RAGE/TLRs-NF-*κ*B pathway in resveratrol-treated DCM mice model.

Therefore, in this present study, we hypothesize that resveratrol administration may inhibit HMGB 1-RAGE/TLRs-NF-*κ*B pathway, oxidative stress, inflammation, and fibrosis in streptozocin- (STZ-) induced diabetic hearts.

## 2. Methods

### 2.1. Animals and Procedures

The experimental and feeding protocols were approved by and in accordance with the laws and regulations controlling experiments on live animals in China and the Asian Convention for the Protection of Vertebrate Animals Used in Experimental and Other Scientific Purposes. The animal experiments were approved by the Medical Ethics Committee of Drum Tower Hospital affiliated to Nanjing University Medical School. Mice (male) were purchased from Model Animal Research Center of Nanjing University. Mice were randomly assigned into four age- and weight-matched groups containing eight mice each. Diabetic mice were induced by consecutive intraperitoneal administration of STZ (40 mg/kg/day, Sigma-Aldrich, St. Louis, MO, USA) for 5 days. For glucose levels measurements, blood was withdrawn from mouse tail-vein and blood glucose was detected with a blood glucose monitor, while plasma insulin was not measured in this experiment. Nonfasting mice with a blood glucose level above 13.9 mM at 3 days after the last STZ injection were considered as diabetic mellitus. Just one STZ-injected mouse (about 11% of the mice injected with STZ) was excluded from the study as the blood glucose did not reach 13.9 mM.

To be similar to clinic, the administration of resveratrol was performed after one-month induction of diabetes as previously reported [6, 20]. One month after induction of diabetes, mice were treated with resveratrol (5 or 25 mg/kg/day via intragastrical administration, Sigma-Aldrich, St. Louis, MO, USA) for another two months. Mice treated with saline containing 0.5% CMC (vehicle) were used as control group. The dose of resveratrol was adjusted every week based on any change in body weight during the whole period of study. Normal chow and water were freely available. Experimental studies showed the dose of intragastrically administered resveratrol leading to cardioprotection range between 2.5 mg/kg/day and 20 mg/kg/day [7, 10, 21], so the resveratrol doses of 5 and 25 mg/kg/day were selected in this experiment.

Changes in body weight and blood glucose were recorded weekly until sacrifice, while 24 h urine output was detected at the end of this experiment.

### 2.2. Masson's Trichrome Staining

Heart samples were fixed in 4% paraformaldehyde and were imbedded in paraffin according to the standard procedures. 4 *μ*m serial sections were longitudinally cut from subepicardial to subendocardial layer and subjected to Masson's trichrome staining. Three sections with 10 fields for one sample were analyzed. Masson's trichrome staining was used to evaluate the extent of fibrosis in all sections. Myocardial cells were stained red and collagenous fibers were stained blue. Collagen volume fraction (CVF) was detected to measure the percentage of heart sectional area comprised of fibrous tissue using Image Pro Plus software. The calculation formula of CVF in each view of the slice is CVF = collagen area/total area × 100%.

### 2.3. Dihydroethidium (DHE) Staining

In this study, DHE staining was used to evaluate superoxide expression. The heart sections were incubated with 2 *μ*m/mL DHE dye (Beyotime Institute of Biotechnology, Haimen, China) for 30 min at 37°C protected from light. Fluorescence pictures were obtained using a fluorescence microscope. Red staining indicating oxidative stress was quantified in 3 randomly selected regions in heart sections in 4 animals/group using Image Pro Plus software.

### 2.4. Western Blot

Left ventricular tissues were homogenized in RIPA buffer containing a 1 : 100 dilution of protease inhibitor (Sigma-Aldrich, St. Louis, MO, USA), and the supernatants were used for western blot after centrifugation. Equal protein samples were subjected to SDS-PAGE. Proteins were transferred electrophoretically to polyvinylidene difluoride membranes (Merck Millipore, Billerica, MA, USA). Then the blots were blocked and incubated with primary antibodies as follows: anti-HMGB 1 (1 : 2000, Bioworld Technology, Inc., St. Louis, MN, USA), anti-RAGE (1 : 500, Abcam, Cambridge, MA, UK), anti-TLR4 (1 : 500, Bioworld Technology, Inc., St. Louis, MN, USA), anti- NF-*κ*B (1 : 1000, Bioworld Technology, Inc., St. Louis, MN, USA), anti-p66shc (1 : 2000, Santa Cruz Biotechnology, Inc., Dallas, TX, USA), anti-gp91phox (1 : 1000, Santa Cruz Biotechnology, Inc., Dallas, TX, USA), anti-p47phox (1 : 500, Bioworld Technology, Inc., St. Louis, MN, USA), anti-TNF-*α* (1 : 500, Bioworld Technology, Inc., St. Louis, MN, USA), and anti-iNOS (Cell Signaling Technology, Danvers, MA, USA). Anti-*β*-actin antibody (1 : 2000, Santa Cruz Biotechnology, Inc., Dallas, TX, USA) was used as the internal control. After four washes in TBST, the blots were incubated with horseradish peroxidase-conjugated secondary antibodies. The washes were repeated, and the membranes were then treated with Super Signal Substrate Western Blotting Reagent (Merck Millipore, Billerica, MA, USA). The bands were quantified using Bio-Rad Quantity One imaging software.

### 2.5. Measurement of Serum HMGB-1 Levels

At the end of this investigation, the mice were anaesthetized and sacrificed by cervical decapitation; the blood was collected for serum separation. Then serum HMGB-1 concentrations were detected using a mouse HMGB-1 ELISA kit (Uscn Life Science Inc., Wuhan, China) according to the manufacturer's instructions.

### 2.6. Isolation of Monocytes from Mouse Bone Marrow

Bone marrow-derived monocytes were isolated as previously described [22]. Briefly, bone marrow was collected from the femurs and tibias and was flushed with cold PBS. After being resuspended in red blood cell lysing buffer, the cells were centrifuged and the monocytes were collected by Ficoll Paque PLUS (GE Healthcare Life Sciences, Little Chalfont, Buckinghamshire, UK) density gradient centrifugation of bone marrow.

### 2.7. Statistical Analysis

One-way analysis of variance (normally distributed) or Kruskal-Wallis test (nonnormally distributed) was used for four groups in this investigation. Values of *p* < 0.05 were considered significant, and all *p* values were two-sided. Analyses were performed with SPSS 21.0.

## 3. Results

### 3.1. Resveratrol Treatment Partially Regulated Biological Parameters in Diabetic Mice

Table 1 details the characteristics of four groups of mice at the end of the experimental period. DM mice exhibited markedly elevated blood glucose levels compared with normal mice and such alterations were partially reversed by resveratrol at a dose of 5 mg/kg/day or 25 mg/kg/day. The 24 h urine volume is an important characteristic of type 1 diabetes, so it is also recorded in this experiment. As shown in Table 1, 24 h urine volume in DM group was increased to 11.63 times as compared with N group, while it was reduced in both DMR5 and DMR25 groups compared to DM group. In addition, the reduced body weight induced by DM was significantly reversed by resveratrol at a dose of 25 mg/kg/day.

### 3.2. Resveratrol Decreased HMGB 1 in Serum and in Bone Marrow-Derived Monocytes from Diabetic Mice

**Figure 1:**
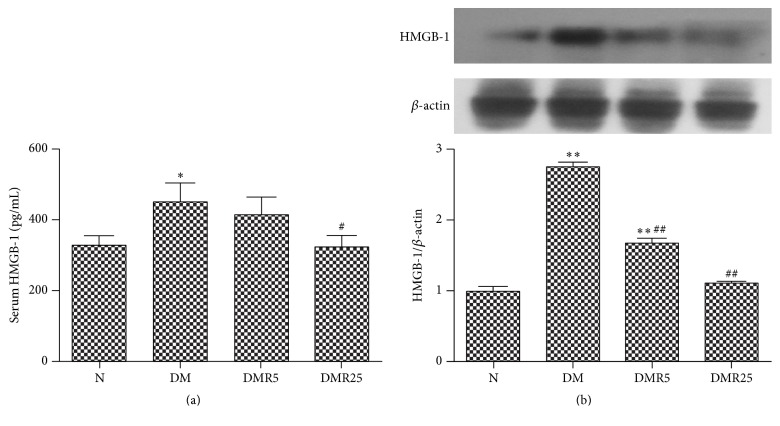
Resveratrol regulated HMGB 1 in serum and monocytes. (a) Resveratrol reduced the secretion of HMGB 1 to serum. (b) Resveratrol downregulated HMGB 1 expression in monocytes. Number of animals: 8 per group. ^*∗*^ *p* < 0.05 versus N group; ^*∗∗*^ *p* < 0.01 versus N group; ^#^ *p* < 0.05 versus DM group; ^##^ *p* < 0.01 versus DM group. HMGB 1: high mobility group box-1; N: normal mice; DM: diabetic mice; DMR5: diabetic mice with 5 mg/kg/day resveratrol treatment; DMR25: diabetic mice with 25 mg/kg/day resveratrol treatment.

It was observed that HMGB 1 in serum was higher in DM group as compared to N group, while it was normalized with resveratrol at a dose of 25 mg/kg/d (Figure 1(a)). As shown in Figure 1(b), the increased intracellular HMGB 1 protein in monocytes was abrogated by resveratrol treatment (Figure 1(b)).

### 3.3. Resveratrol Inhibited HMGB 1-Mediated Signaling Pathway in Diabetic Hearts

**Figure 2:**
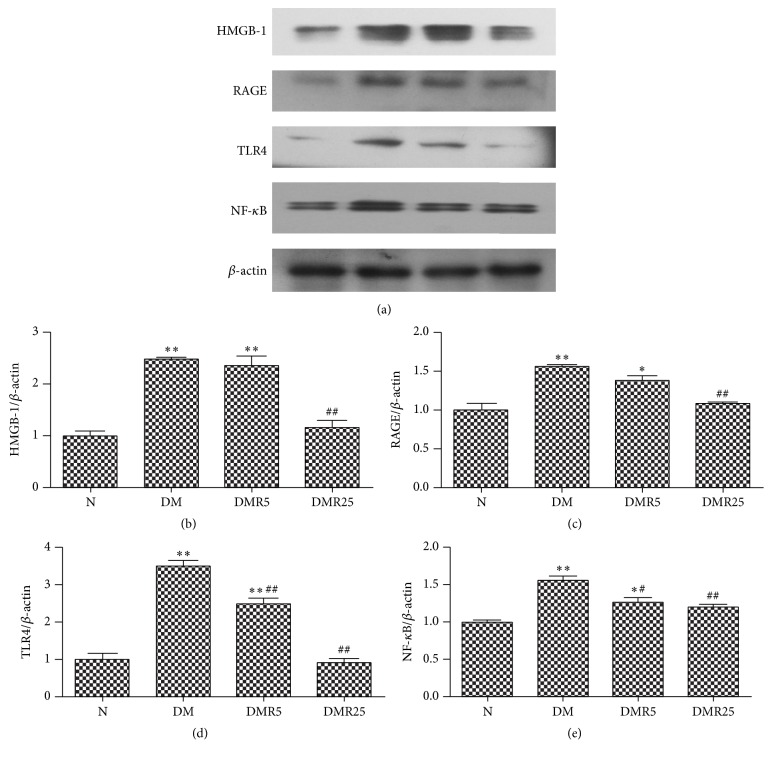
Western blot analysis of HMGB 1, RAGE, TLR4, and NF-*κ*B proteins in hearts of four groups. (a) Representative immunoblots of HMGB 1, RAGE, TLR4, and NF-*κ*B in four groups. ((b)–(e)) Protein analysis of HMGB 1, RAGE, TLR4, and NF-*κ*B. Number of animals: 8 per group. ^*∗*^ *p* < 0.05 versus N group; ^*∗∗*^ *p* < 0.01 versus N group; ^#^ *p* < 0.05 versus DM group; ^##^ *p* < 0.01 versus DM group. HMGB 1; high mobility group box-1; RAGE: receptor for advanced glycation end products; TLR4: toll-like receptor 4; NF-*κ*B: nuclear factor *κ*B; N: normal mice; DM: diabetic mice; DMR5: diabetic mice with 5 mg/kg/day resveratrol treatment; DMR25: diabetic mice with 25 mg/kg/day resveratrol treatment.

To investigate the role of HMGB 1 in cardioprotective effect of resveratrol in DCM, HMGB 1 and its downstream effectors were examined by western blot. Similar to its protein level in serum and monocytes, increased expression of HMGB 1 in diabetic hearts was inhibited by treatment with 25 mg/kg/day resveratrol (Figure 2(b)). RAGE and TLR4, as receptors of HMGB 1, were both upregulated in STZ-induced diabetic mice; however, treatment with 25 mg/kg/day resveratrol reversed these changes in DM mice. In addition, treatment of diabetic mice with resveratrol at a low dose of 5 mg/kg/day significantly attenuated DM-induced HMGB 1 expression in hearts. As HMGB 1 exhibited its effects in cardiovascular system by binding to RAGE/TLRs, resulting in activation of NF-*κ*B, the expression of NF-*κ*B was also tested by western blot in this study. As illustrated in Figure 2(e), compared with the N group, the DM group showed significantly higher NF-*κ*B expression, which was suppressed in DMR5 and DMR25 groups.

### 3.4. Resveratrol Suppressed Oxidative Stress in Diabetic Hearts

**Figure 3:**
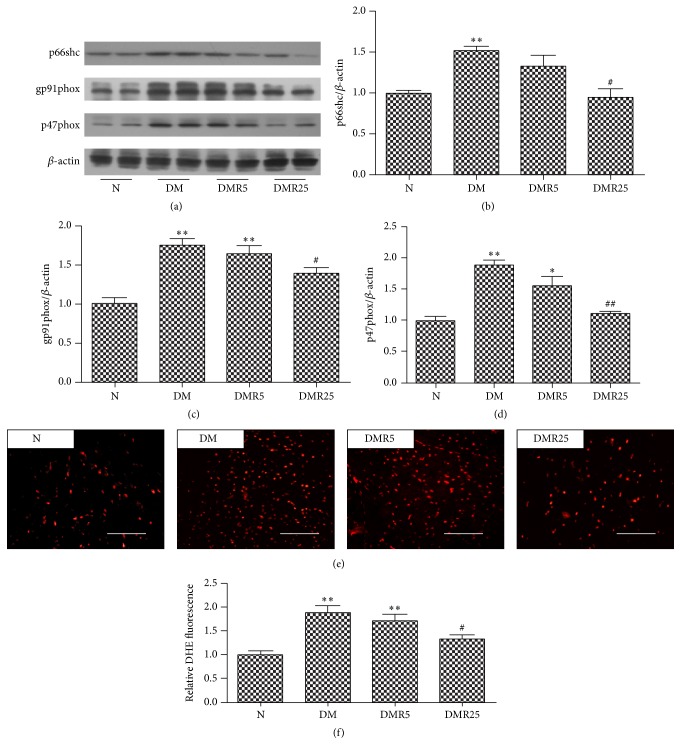
Effects of resveratrol on DM-induced oxidative stress. ((a)–(d)) Western blot analysis of p66shc, p47phox, and gp91phox levels in four groups. ((e), (f)) Observation of ROS by DHE staining in heart sections. Number of animals: 8 per group. ^*∗*^ *p* < 0.05 versus N group; ^*∗∗*^ *p* < 0.01 versus N group; ^#^ *p* < 0.05 versus DM group; ^##^ *p* < 0.05 versus DM group. Bar = 50 *μ*m. DHE: dihydroethidium; N: normal mice; DM: diabetic mice; DMR5: diabetic mice with 5 mg/kg/day resveratrol treatment; DMR25: diabetic mice with 25 mg/kg/day resveratrol treatment.

Resveratrol demonstrated its cardioprotection via inhibition of oxidative stress, which was considered to be associated with HMGB 1. So western blot was performed to analyze expressions of p66shc, p47phox, and gp91phox, which are all oxidases contributing to oxidative stress. As shown in Figure 3(b), the expression of p66shc was higher in DM group than that in N group, while its protein level in DMR25 group was significantly lower than that of DM group. Moreover, upregulated expressions of NADPH oxidase subunits p47phox and gp91phox induced by DM were significantly attenuated by treatment of both 5 mg/kg/day and 25 mg/kg/day resveratrol.

To further corroborate the effect of resveratrol on oxidative stress in diabetes, we detected ROS production by DHE staining. As shown in Figure 3(e), treatment with resveratrol at a single dose of 25 mg/kg/d protected the diabetic mice from oxidative stress as indicated by decreased red signaling.

### 3.5. Resveratrol Ameliorated Cardiac Fibrosis and Inflammation in Diabetic Heart

**Figure 4:**
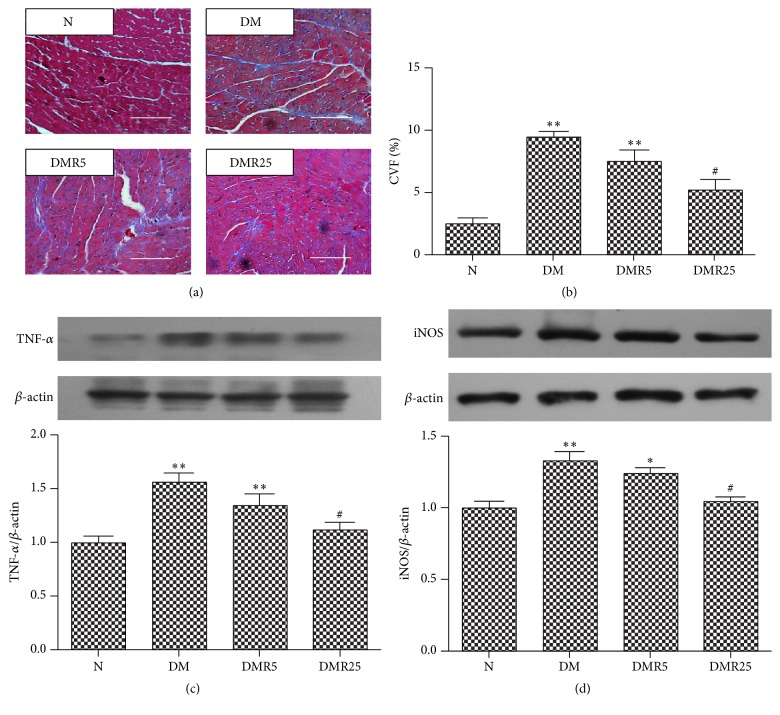
Resveratrol attenuated myocardial fibrosis and inflammation in the hearts of experimental type 1 diabetic mice. (a) Representative Masson's staining in four groups. (b) Quantitative analysis of CVF in four groups. (c) Representative western blot of TNF-*α* expression. (d) Representative western blot of iNOS level. Number of animals: 8 per group. ^*∗*^ *p* < 0.05 versus N group; ^*∗∗*^ *p* < 0.01 versus N group; ^#^ *p* < 0.05 versus DM group. Bar = 50 *μ*m. CVF: collagen volume fraction; TNF-*α*: tumor necrosis factor-*α*; iNOS: inducible nitric oxide synthase; N: normal mice; DM: diabetic mice; DMR5: diabetic mice with 5 mg/kg/day resveratrol treatment; DMR25: diabetic mice with 25 mg/kg/day resveratrol treatment.

In this section, we detected changes of myocardial fibrosis and inflammation in hearts of different groups. Masson's staining was used to identify the degree of myocardial fibrosis. The collagen volume fraction (CVF) increased significantly compared with N group; however, 25 mg/kg/day resveratrol reduced the degree of myocardial fibrosis in diabetic hearts (Figures 4(a) and 4(b)). In this investigation, we found enhanced expressions of TNF-*α* and iNOS in DCM models, while treatment with resveratrol at a dose of 25 mg/kg/day significantly ameliorated these expressions (Figures 4(c) and 4(d)).

## 4. Discussion

The present study shows that treatment with resveratrol can prevent HMGB1/RAGE/TLR4/NF-*κ*B pathway, oxidative damage, myocardial fibrosis, and inflammation in STZ-induced type 1 diabetic hearts.

Over the last 5 years, growing evidence considered HMGB 1 as a key factor in promoting and maintaining diabetic complications [12, 23, 24]. Recently, Wang and Delucchi found that HMGB 1 was diffusely expressed in the myocardium of diabetic mice [10, 16]. The findings are in agreement with our results provided in Figure 2(b). HMGB 1, a proinflammatory factor, is secreted from immune cells to serum under some conditions. So we detected the level of HMGB 1 in serum and monocytes from diabetic mice. Interestingly, a significant increase of HMGB 1 in serum and monocytes was observed in DM group, and the elevated HMGB 1 expression was normalized by resveratrol treatment. In favor of this deduction, Yang et al. recently found that resveratrol reduced lipopolysaccharide-induced expression of HMGB 1 in murine macrophage-like RAW264.7 cells [25]. Indeed, the results from the present study showed that HMGB 1 may be a potential target for resveratrol in DCM.

Oxidative stress has been recognized as an important link between DM and DCM. NADPH oxidase is considered to be a major source of ROS and plays a critical role in diabetic complications. By detecting the expressions of p47phox and gp91phox with western blot, NADPH oxidase activity was elevated in hearts of STZ-induced type 1 DM, whereas resveratrol-treated DM mice revealed ameliorated NADPH oxidase activity. This notion is strongly supported by a previous study suggesting the decreased NADPH activity of resveratrol treatment in type 2 DM [21]. Consistent with a previous observation [26], p66shc, another oxidative stress related protein, was also upregulated in STZ-induced type 1 diabetic hearts. Interestingly, the main new finding of this study was that the increased expression of p66shc in diabetic hearts was reversed by treatment of resveratrol.

**Figure 5:**
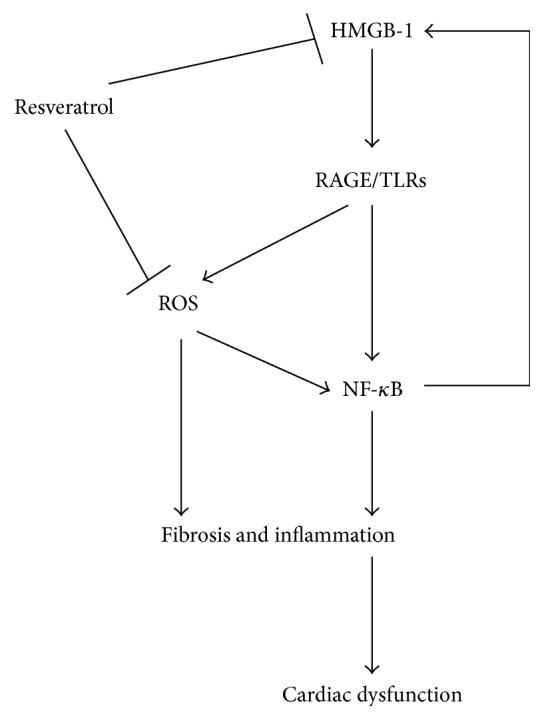
Schematic diagram for the possible mechanisms of the cardioprotection of resveratrol in diabetic cardiomyopathy. By binding to RAGE or TLRs, HMGB 1 stimulates ROS production and activation of NF-*κ*B, which triggers inflammation and fibrosis in diabetic hearts. Increased ROS production and inflammation may be responsible for upregulated HMGB 1 with a positive feedback. Treatment with resveratrol attenuates genesis of ROS and HMGB 1 to prevent cardiac dysfunction in diabetes.

As mentioned above, HMGB 1 as well as oxidative stress was increased in DM, thus, it could speculate that HMGB 1 might be linked to oxidative stress in DM. H_2_O_2_ converted from superoxide stimulated macrophages and monocytes to actively release HMGB 1 [27]. Once released, extracellular HMGB 1 might cause activation of NADPH oxidase as well as increased ROS production in a TLR4-depended pathway [28]. Recently, we have previously found that HMGB-1 was involved in diabetes-induced oxidative stress in endothelial progenitor cells [22]. Together, these findings indicate that HMGB 1 might be an inducer of oxidative stress or an effector of oxidative stress in DCM (Figure 5), which needs to be confirmed by further studies.

Over the last decade, AGE/RAGE was considered to be a key element contributing to diabetic complications. HMGB 1, another ligand of RAGE, was also involved in diabetes-induced myocardial fibrosis, where they reported that HMGB 1 inhibition improved cardiac function and remodeling in diabetic mice [16]. Besides that, HMGB 1 was shown to be associated with hepatic fibrosis [18] and renal fibrosis [29]. The underlying mechanisms were poorly understood, although several studies demonstrated that the profibrotic effect of HMGB 1 might be due to binding to its receptors such as RAGE, TLR4, and TLR2 [18, 30]. In line with our investigation, several reports indicated that RAGE, NADPH oxidase, and fibrosis were enhanced in diabetic hearts [31, 32]. In this investigation, TLR4 expression and ROS production were upregulated in DM mice compared to normal mice. Consistent with the findings, TLR4 as well as ROS was upregulated in diabetic hearts, suggesting the critical role of ROS/TLR4 in DCM [33, 34]. More importantly, the expression of HMGB 1/RAGE/TLR4 and cardiac fibrosis were attenuated by administration of resveratrol, supporting the previous observation that resveratrol inhibited RAGE and TLR 4 dependent pathways [35, 36]. Cardiac fibroblasts, the main cells involved in myocardial fibrosis, were considered to promote fibrosis via proliferation, collagens expression, and differentiation into myofibroblast phenotype in DCM [6, 37]. Several studies indicated that fibroblasts were the source of HMGB 1 [16, 38, 39]. Besides, damage-associated molecular patterns (including HMGB 1) have been shown to provoke fibroblast activation and trigger myocardial fibrosis [16, 39], suggesting that the crosstalk between HMGB 1 and cardiac fibroblasts might play an important role in myocardial fibrosis in DCM. Taken together, the data demonstrated that inhibition of ROS/HMGB 1/RAGE/TLR4 pathway and HMGB 1-mediated activation of fibroblasts are, at least in part, responsible for the antifibrotic effect of resveratrol in DCM.

DM is an inflammatory disease, and inflammation plays an important role in the pathogenesis of DCM. The present study indicated that treatment with resveratrol provided anti-inflammatory effect and limited HMGB 1 expression in diabetic hearts, suggesting that inhibition of inflammatory response in diabetic hearts might be linked to normalized HMGB 1 secretion.

HMGB 1 could evoke the activation of proinflammatory pathways in cardiovascular system via binding to RAGE [40–43]. In this investigation, we found that both HMGB 1 and RAGE were increased in diabetic compared to normal animals, while treatment with resveratrol could inhibit the changes. The results supported a previous observation that resveratrol abrogated DM-induced RAGE expression [35]. In addition, TLR4, another receptor of HMGB 1 induced by diabetes, was also attenuated by supplement of resveratrol. More and more evidence revealed the important role of TLR4-mediated signaling pathway in inflammation. Recently, it was shown that HMGB 1/TLR4 signaling contributed to inflammation in hepatic ischemia/reperfusion injury [44]. However, Mudaliar et al. suggested TLR4 as an important mediator for inflammation in high glucose-induced endothelial cells [45]. Furthermore, the TLR4-mediated inflammation was inhibited by resveratrol in various diseases [36, 46, 47]. Thus, taking the present results together, resveratrol may prevent inflammation in DCM via inhibition of HMGB 1/TLR4 pathway. NF-*κ*B is a major downstream molecule of RAGE/TLR4 as well as an important transcriptional factor of various inflammatory mediators. As expected, NF-*κ*B expression was suppressed in diabetic mice by treatment of resveratrol. Similar to HMGB 1 and NF-*κ*B, the expressions of TNF-*α* and iNOS in hearts were both increased in DM group, but treatment with resveratrol restored them to normal in DMR25 group, which is in agreement with a previous investigation [21]. In addition, these results also supported previous observation that resveratrol exhibited its cardioprotective effect or cerebroprotective effect via inhibition of TNF-*α* related inflammation [48, 49], indicating TNF-*α* as an important inflammatory factor in DCM. Currently, increasing studies have showed that HMGB 1 could promote proinflammatory expression including TNF-*α*. For example, HMGB 1 was shown to mediate high glucose-induced TNF-*α* expression in cardiomyocytes [12]. However, Chen et al. [50] found that direct suppression of TNF-*α* activity partially attenuated HMGB 1 release from macrophages, suggesting that some inflammatory cytokines in turn triggered the activation of many immune cells and enhanced HMGB 1 expression [51]. Namely, HMGB 1 can function as a proinflammatory cytokine, which in turn forms a positive feedback loop, thus exaggerating inflammatory state in DM (Figure 5). Thus, in the present study, it is worth mentioning that HMGB 1 might be an inducer or an effector of TNF-*α* and iNOS in DCM. In conclusion, the results demonstrated that resveratrol ameliorated upregulated HMGB 1 release and related proteins, accompanied with ROS genesis, myocardial fibrosis, and inflammation in diabetic hearts, though our study raises more questions than answers regarding the relationship and crosstalk between them. Taken together, we present HMGB 1-mediated signaling pathway as a novel therapeutic strategy of resveratrol in treatment of DCM.

## Figures and Tables

**Table 1:** Resveratrol modulated blood glucose, 24 h urine output, and body weight in diabetes mellitus.

Groups	N	DM	DMR5	DMR25
Blood glucose (mmol/L)	7.4 ± 0.5	27.2 ± 2.8^*∗∗*^	24.6 ± 1.2^#^	20.9 ± 1.3^##^
24 h urine output (mL)	0.98 ± 0.24	11.4 ± 2.3^*∗∗*^	8.0 ± 1.9^##^	6.2 ± 1.72^##^
Body weight (g)	30.4 ± 1.5	23.0 ± 1.5^*∗∗*^	24.6 ± 1.2	25.2 ± 1.6^#^

Data are expressed as mean ± SD. Number of animals: 8 per group. ^*∗∗*^ *p* < 0.01 versus N group. ^#^ *p* < 0.05 versus DM group. ^##^ *p* < 0.01 versus DM group. N: normal mice; DM: diabetic mice; DMR5: diabetic mice with 5 mg/kg/day resveratrol treatment; DMR25: diabetic mice with 25 mg/kg/day resveratrol treatment.
